# Modified Coronally Advanced Tunnel Technique With Porcine Dermal Matrix for Recession Treatment: 12‐Month Follow‐Up

**DOI:** 10.1002/cre2.70199

**Published:** 2025-08-22

**Authors:** Erik Würflein, Sebastian Ollinger, Anton Sculean, Kirstin Vach, Victoria Constanze Landwehr, Katja Nelson, Betül Dursun, Susanne Nahles, PD Gerhard Iglhaut, Tobias Fretwurst

**Affiliations:** ^1^ Department of Oral and Maxillofacial Surgery/Translational Implantology, Faculty of Medicine Medical Centre – University of Freiburg Freiburg Germany; ^2^ Department of Periodontology University of Bern Bern Switzerland; ^3^ Institute of Medical Biometry and Statistics, Faculty of Medicine and Medical Centre University of Freiburg Freiburg Germany; ^4^ Department of Oral and Maxillofacial Surgery, Berlin Institute of Health, Corporate Member of Freie Universität Berlin, Charité – Faculty of Medicine Berlin Humboldt‐University of Berlin Berlin Germany

**Keywords:** coronally advanced tunnel technique, digital measurement, gingival recession, porcine dermal matrix, recession reduction, recession type, root coverage

## Abstract

**Objectives:**

To assess the efficacy of the modified coronally advanced tunnel technique (MCAT) with a porcine dermal matrix (PDM) after a 12‐month follow‐up. There are no other Clinical trials evaluating a PDM over the period of 12 months.

**Material and Methods:**

Patients with recession type (RT) 1 and RT 2 gingival recessions were treated with the MCAT and a novel PDM. Plaster casts (preoperative and 12 months postoperative) were scanned using a 3Shape Lab Scanner E3. The resulting STL files were imported and superimposed in the open‐source software GOM Inspect for analysis. Measurements included recession depth, mean root and total root coverage (mRC and cRC), mean recession reduction (mRR), and gingival thickness. Statistical analysis was performed using mixed linear models.

**Results:**

A total of 77 teeth (19 patients) were included in the study. Healing was uneventful in all patients. The mean preoperative recession depth was 1.26 mm ± 0.86 mm. mRC was 69.47% ± 61.90%, cRC was 29.79%, mRR was 0.87 ± 0.83 mm, and gingival thickness gain was 0.23 ± 0.24 mm, with comparable results for RT 1 and RT 2. Neither tooth type nor jaw type had any effect on root coverage.

**Conclusions:**

The modified coronally advanced tunnel technique in combination with the analyzed porcine dermal matrix demonstrated stable results for root coverage and gingival thickness after 12 months of follow‐up.

**Trial Registration:** Deutsches Register Klinischer Studien/German Clinical Trial Register (DRKS); DRKS00023201.

**Abbreviations**
CEJ: cemento–enamel junction
cRC: total root coverage
CTG: connective tissue graft
DRKS: Deutsches Register Klinischer Studien
KTW: keratinized tissue width
MCAT: modified coronally advanced tunnel
mRC: mean root coverage
mRR: mean recession reduction
PDM: porcine dermal matrix
RCT: randomized controlled trial
RT: recession type

## 1 Introduction

Gingival recession, which is a common condition characterized by the exposure of the tooth root due to the apical displacement of the gingival margin, poses challenges to both periodontal health and esthetics (Chambrone et al. 2019). Gingival recessions can lead to discomfort and pain, especially during activities like tooth brushing (Miranda‐Rius et al. 2018). Orthodontic treatment, although beneficial, has been associated with an increased prevalence of gingival recession, particularly in cases involving proclination of the lower incisors (Joss‐Vassalli et al. 2010). Furthermore, a thin gingival phenotype can amplify the risk of gingival recession and even more when combined with the presence of plaque‐induced gingivitis and chronic periodontitis (Merijohn 2016). However, patients with a thick gingival phenotype can also be affected by gingival recessions (Kassab and Cohen 2003). Gingival recessions are classified using the RT system, which helps to distinguish different levels of recession, based on the presence or absence of interproximal attachment loss (Zucchelli and Mounssif 2015). RT 1 indicates gingival recessions without interproximal attachment loss, RT2 indicates recessions with interproximal attachment loss equal to or less than the buccal site, and RT3 indicates recessions with interproximal attachment loss exceeding the buccal site (Cairo et al. 2011). The ideal treatment for gingival recessions involves root coverage and soft tissue thickening through gingival grafting or guided tissue regeneration (Cairo et al. 2014). Among the various surgical procedures, the modified coronally advanced tunnel technique (MCAT) has been established as an effective approach for treating multiple gingival recessions (Zucchelli and Mounssif 2015; Carvalho et al. 2006; Kassab et al. 2010; Aroca et al. 2013; Sculean et al. 2014; Sculean et al. 2016; Sculean et al. 2017). Autologous palatal connective tissue grafts combined with bilaminar techniques to ensure graft vascularization are considered the gold standard for achieving predictable results in root coverage procedures (Cairo et al. 2008). However, the use of autologous connective tissue grafts may have drawbacks such as donor site morbidity (Vincent‐Bugnas et al. 2021). Therefore, matrices have been described as a promising alternative to autologous connective tissue grafts. Studies have shown that materials such as acellular allograft dermis (Aichelmann‐Reidy et al. 2001), xenogenic collagen matrices (Moustafa et al. 2022), and xenogeneic collagen matrices (De Annuntiis et al. 2022) can provide successful outcomes in terms of root coverage and esthetics. However, xenogenic collagen matrices achieve less favorable results compared to autologous grafts (Aroca et al. 2013; Cosgarea et al. 2016). Comparative studies using porcine dermal matrices (PDM) with MCAT to cover Miller Classes I, II, and III recessions reported a mean root coverage (mRC) of 53.2%–73.2%, compared to 80.6%–90% for autologous connective tissue grafts. Complete root coverage was obtained in 20% to 43% of PDM cases, compared to 40% to 59% with autologous grafts (Aroca et al. 2013; Vincent‐Bugnas et al. 2021; Cosgarea et al. 2016; Pietruska et al. 2019). In the past, the clinical measurement of soft tissue changes has been performed using a periodontal probe in relation to the cemento–enamel junction (CEJ) (Kuralt et al. 2021). New digital techniques using 3D superimposition of preoperative and postoperative data have been suggested (Rebele et al. 2014) and successfully performed (Iglhaut et al. 2024) by using CEJ‐dependent and ‐independent measuring approaches. However, to facilitate comparison with other clinical studies, in the present study recession depth, mean root, and total root coverage were measured at the CEJ on the digital models. To the best of the authors' knowledge, no 12‐month follow‐up data are currently available for the PDM used. Therefore, the primary objective of this clinical study was to evaluate the outcome of gingival recession coverage using the MCAT in combination with a novel PDM over a 12‐month follow‐up period.

## 2 Materials and Methods

### 2.1 Study Design and Trial Registration

The study was designed as a prospective, single‐arm clinical study and registered at the Deutsches Register Klinischer Studien/German Clinical Trial Register (DRKS, DRKS00023201). It followed the STROBE guidelines (https://www.equator-network.org/reporting-guidelines/strobe/). The study protocol received approval from the ethics committee of the Faculty of Medicine at the University of Freiburg, Germany (No 352/19) and complied with the Declaration of Helsinki of 1975, revised in Fortaleza in 2013. All participants were informed about the study's objectives and details and provided written informed consent.

### 2.2 Participants

From November 2019 to September 2022, patients were enrolled at the Department of Oral and Maxillofacial Surgery, Faculty of Medicine, University of Freiburg, Germany. Those with single or multiple gingival recessions (RT 1 or RT 2) in either the upper or lower jaw underwent root coverage using a PDM (Novomatrix Reconstructive Tissue Matrix; BioHorizons Camlog, Basel, Switzerland) employing the MCAT (Aroca et al. 2010); Zuhr et al. 2021). All participants were of legal age and maintained good oral hygiene (full mouth plaque score < 20%). Exclusion criteria comprised gingival recessions classified as Miller class IV or RT 3, periodontitis, systemic disorders (such as diabetes, immunosuppression, cardiovascular diseases, irradiation, and chemotherapy), disrupted or altered bone metabolism, and parafunctional habits. Nicotine users and individuals concurrently involved in other studies were ineligible for participation in this study.

### 2.3 Intervention

**Figure 1:**
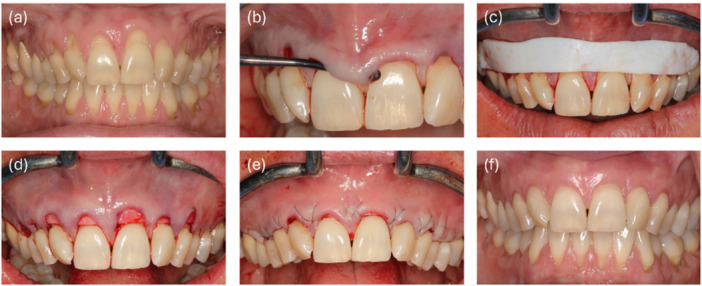
(a) Preoperative condition, (b) preparation of a continuous subperiosteal tunnel, (c) adjustment of the PDM, (d) placement of the PDM into the tunnel, (e) fixation of the PDM with loop sutures, and (f) clinical outcome 12 months postoperatively.

The root coverage procedure was conducted by a single proficient surgeon (GI) and performed as described before (Iglhaut et al. 2024). Briefly, each patient underwent professional tooth cleaning before surgery and had alginate casts (Pluralgin NF, Pluradent GmbH & Co. KG, Offenbach am Main, Germany) made three to 5 days before surgery. Oral antibiotic therapy (amoxicillin 1000 mg three times daily) was initiated the evening before surgery and continued for 10 days. This extended regimen was based on institutional protocol for mucogingival procedures involving biomaterials and regenerative adjuncts such as porcine dermal matrix (PDM) and leukocyte‐ and platelet‐rich fibrin (L‐PRF). In tunneling techniques, where subperiosteal placement limits visibility and decontamination, the risk of microbial contamination is elevated. Although this dosage exceeds typical regimens used for standard odontogenic infections, it was intended to reduce the risk of postoperative infection and to promote optimal integration and healing of the graft material within the soft tissue. Patients received 600 mg ibuprofen immediately before the procedure. Venous blood was drawn for the extraction of leukocyte‐ and platelet‐rich fibrin (L‐PRF, IntraSpin, Intra‐Lock, Boca Raton, FL, USA). Local anesthesia (Artinestol 1:200,000, Merz Dental, Lütjenburg, Germany) was administered. The exposed root surfaces were cleaned and smoothed using a curette (Younger‐Good curette 7/8, HuFriedy, Chicago, USA), followed by conditioning with EDTA gel (PrefGel, Straumann, Basel, Switzerland) for 2 min. This step, which is frequently used for conditioning of the root surface on periodontally diseased roots and in root coverage, forms a surface suitable for cell attachment, growth, migration, and differentiation (Zhan et al. 2021). Subsequently, surgical root coverage was performed using the minimally invasive MCAT (Figure 1).

The gingiva was separated from the bone surface with a precise intrasulcular incision using a microblade (Key Dent, American Dental Supply, Vaterstetten, Germany). Tunneling instruments (Iglhaut Tunnel Set, HuFriedy, Chicago, IL, USA) were employed to gently create a continuous subperiosteal tunnel, ensuring the flap tissue and papillae remained intact. In cases of extremely thin gingival phenotype, a papilla base incision followed by a precise supraperiosteal preparation was conducted to mitigate the risk of soft tissue perforation. The PDM underwent a thorough cleansing in sterile saline for at least 5 min before being treated with leukocyte‐ and platelet‐rich fibrin and applied onto the prepared site using a curette 7/8 (Younger‐Good, Hu‐Friedy Group, Frankfurt am Main, Germany). According to described results in in‐vitro studies, L‐PRF was used (Lin et al. 2020). The matrix was securely placed within the tunnel and fixed in position. Loop sutures (6‐0 Seralene, Serag‐Wiessner, Naila, Germany) were utilized to displace the tunnel tissue coronally to the enamel‐cement interface. The suture starts lingually and passes approximately without tissue fixation. At the vestibular aspect, the suture captures the PADM, followed by the wound flap. Next the needle perforates the soft tissue at the papilla base to the lingual aspect and coming back buccally at the other approximal space. Again, the suture captures the PADM, followed by the wound flap and going back lingually through the papilla base. Finally, the suture is knotted around the tooth at the lingual aspect. An extraoral tape (Fixomull stretch, BSN medical, Hamburg, Germany) was administered for 5 days post‐surgery to prevent swelling and facilitate stabilization. Patients were instructed for physical rest and utilize an oral rinse (Salviathymol N, Meda Pharma, Radebeul, Germany) twice daily for 3 weeks. Oral hygiene measures in the treated area were restricted for 1 week. By using a soft tooth brush for the careful reduction of biofilm. Follow‐up evaluations were conducted at 1‐, 10‐, and 30 days postoperatively to monitor for complications such as infection, hematoma, postoperative pain, nerve injury, wound dehiscence, duration of analgesic use, and overall satisfaction with the root coverage procedure. Sutures were removed 4 weeks after surgery, and clinical parameters were assessed at the 12‐month mark. The sling sutures used show no signs of irritation after 4 weeks. Using 6‐0 Polypropylene or 6‐0 Polyvinylidenefluoride suture material avoids plaque adhesion and irritation during oral hygiene. Therefore, the patients can start careful oral hygiene with special soft teethbrushes after 1 week without irritating the suture stability and avoiding healing nor tissue irritation. Some studies even suggest suture removal after 2–3 months, showing no signs of irritation or tissue reaction (Allen 2010).

### 2.4 STL File Acquisition and 3D Superimposition

STL file acquisition and 3D Superimposition were performed as previously described (Iglhaut et al. 2024). In brief, plaster casts, made from alginate impressions (Pluralgin NF, Pluradent GmbH & Co. KG, Offenbach am Main, Germany), were anonymized and converted into digital format using the 3Shape Lab Scanner E3 (3Shape, Copenhagen, Denmark). These digital files, in STL format, were then imported into the open‐source software GOM Inspect (Carl Zeiss GOM Metrology GmbH, Braunschweig, Germany) for computerized analysis. The cast digitized 12 months after the procedure was imported as a “mesh” and overlaid onto the “CAD body” using the full arch to align the preoperative and postoperative scans. The software provided an initial alignment, which was further refined manually by selecting tooth surfaces.

### 2.5 Primary Outcome Measure

Root coverage assessment followed the described workflow (Iglhaut et al. 2024). Three evaluations per tooth by two independent investigators, and the mean of these measurements was computed. The following root coverage parameters were employed: recession depth, mean root coverage, total root coverage, mean recession reduction, gingival thickness.

Parameters Dependent on CEJ: For consistency with other clinical studies, measurements for recession depth, mean root coverage, and total root coverage were taken at the CEJ on the digital models.

1. Recession depth:
  The preoperative recession depth was determined as the distance between the deepest point of recession and the CEJ along the tooth axis.
2. Mean root and total root coverage:
  Mean root coverage was calculated by dividing the difference between preoperative and postoperative recession depth by the preoperative recession depth, then multiplying by 100 (Cairo et al. 2009). Complete root coverage was determined by dividing the number of teeth with postoperative covered roots extending to or beyond the CEJ by the total number of teeth, then multiplying by 100 (Di Gianfilippo et al. 2021).

Due to minimal color contrast, the CEJ was not identifiable on the digital models. Hence, it was identified on the plaster models and transferred to the digital models.

Secondary *outcome measure*

Parameters Independent of CEJ:

3. Quantification of reduction of recession depth:
  The reduction in recession depth was measured in millimeters between the points of “preoperative recession” and “postoperative recession” using the “Construct 2‐Point Distance” function in the GOM Inspect software. This reduction in recession depth parameter corresponds to the absolute gain of gingival height (Xue et al. 2022).
4. Gingival Thickness:
  To be able to evaluate the transmitted values as absolute values, equidistant deviation flags were output from the sulcus and at the papilla tips at 1 mm intervals. For the average thickness gain, the average values of the values 2 mm coronal to the sulcus, the sulcus value itself, and the four values apical to the sulcus were calculated. Only in the recession section, three values apical and three values cornal to the sulcus value were evaluated for the average thickness gain.

### 2.6 Statistical Analysis

The sample size for this single‐arm non‐inferiority study was determined based on the mean root coverage parameter with a reference value of 87% (as the mean of publications by Aroca and colleagues) with a non‐inferiority margin of 10%. Assuming a standard deviation of 25%, a total of 66 teeth were required with a power of 90%, an alpha of 5%, and an assumption of a variance inflation factor of 3 if more than one tooth per patient is considered.

Statistical analysis involved computing mean, standard deviation, and the intraclass correlation coefficient. Bar charts and box plots were employed for graphical representation. Linear mixed models with patients as a fixed effect were utilized to analyze differences in various outcome parameters concerning jaw, type of tooth, and recession type. All subsequent pairwise tests were adjusted for multiple testing using Scheffe's method. STATA 17.0 (StataCorp, College Station, Texas, USA) was used for data analysis.

## 3 Results

### 3.1 Baseline Data

25 patients were enrolled in the study, 19 patients (15 females and 4 males) remained for further analysis. Three participants were excluded due to extensive prosthetic treatments during the follow‐up period, which impeded the superimposition. Two patients chose to leave the study voluntarily, and one was excluded due to noncompliance. Postoperative wound healing was complication‐free in all patients. For three patients, recession coverage was performed on both maxillary and mandibular teeth. The mean age of the patients was 46.1 ± 14.9 years. During the study, 77 teeth were surgically treated for recession coverage (Baseline characteristics, Supporting Information).

### 3.2 Preoperative and Postoperative Superimposition Deviation

During the matching process of the preoperative and postoperative models, an average deviation of 0.039 ± 0.007 mm was observed. The deviation for maxillary models was 0.041 ± 0.004 mm on average, and for mandibular models, it was 0.037 ± 0.008 mm.

### 3.3 Preoperative Recession Depth

**Figure 2:**
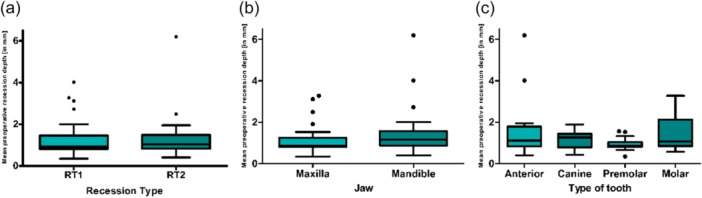
The mean preoperative recession depth was categorized by (a) recession type, (b) jaw type, and (c) tooth type, measured in millimeters. The measurement was taken digitally, from the most apical point of the gingival margin to the cemento–enamel junction (CEJ).

The preoperative baseline revealed an average recession depth of 1.26 ± 0.86 mm (Figure 2). Maxillary recessions had an average depth of 1.09 ± 0.66 mm preoperatively, while mandibular recessions averaged 1.4 ± 0.99 mm (Figure 2). The average recession depth was 1.56 ± 1.37 mm for anterior teeth, 1.12 ± 0.44 mm for canines, 0.95 ± 0.28 mm for premolars, and 1.51 ± 0.87 mm for molars. Recession depth was evenly distributed among the recession types: RT 1 teeth had/showed an average depth was 1.27 ± 0.85 mm, and RT 2 teeth had an average depth of 1.25 ± 0.89 mm (*p* = 0.030).

### 3.4 Mean Recession Coverage After 12 Months

**Figure 3:**
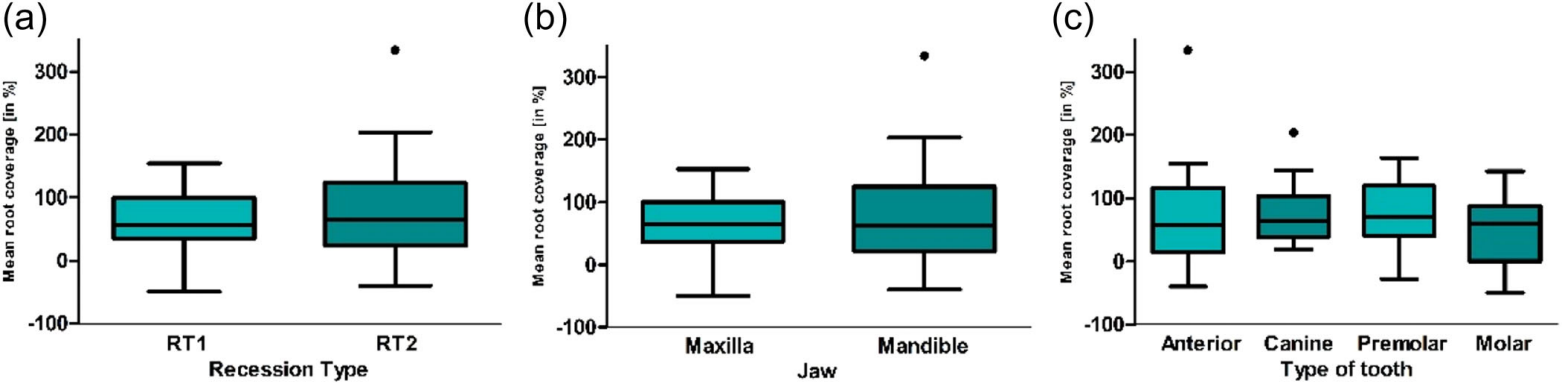
The mean postoperative recession reduction is presented as a percentage (%) based on (a) recession type, (b) jaw type, and (c) tooth type.

The mRC for all teeth was 70.01% ± 61.75%. Teeth with RT 1 had an mRC of 59.37% ± 50.57%, while those with RT 2 achieved an average root coverage of 78.43% ± 68.76% (Figure 3). When considering the jaw, the maxillary teeth had an mRC of 65.24% ± 48.25%, and the mandibular teeth achieved an average root coverage of 73.99% ± 71.43%. The anterior teeth showed a coverage success of 70.98% ± 83.08% mRC. The canines had an mRC of 78.55% ± 52.17% at 12 months postoperation. The premolars achieved an mRC of 76.35% ± 51.04%, and the molars had an mRC of 50.93% ± 57.81%. The mRC did not differ significantly for recession types (*p* = 0.893), jaw type (*p* = 0.250), or tooth type (*p* = 0.331).

### 3.5 Complete Recession Coverage

**Figure 4:**
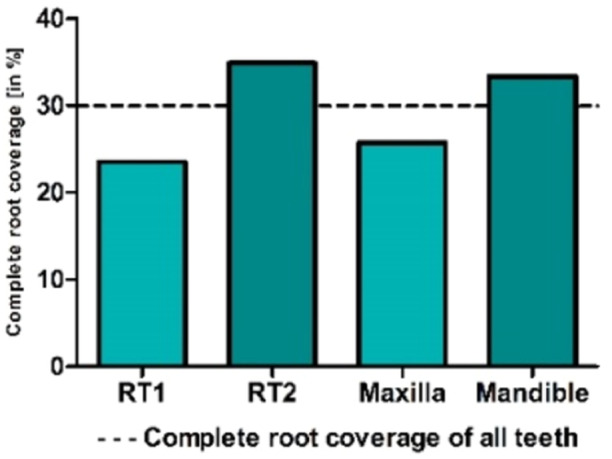
The complete recession reduction is presented as a percentage (%) based on recession type and jaw type relative to the average (dotted line).

cRC was achieved in 29.87% of all teeth (Figure 4). For RT 1, 23.53% of teeth attained complete root coverage, while 34.88% of teeth with RT 2 were fully covered. In the maxilla, 25.71% of teeth achieved complete root coverage, whereas in the mandible, 33.33% of teeth were fully covered (Figure 4). The cRC was not significantly different for the recession type (*p* = 0.966) or jaw type (*p* = 0.270).

### 3.6 Absolute Recession Reduction After 12 Months

**Figure 5:**
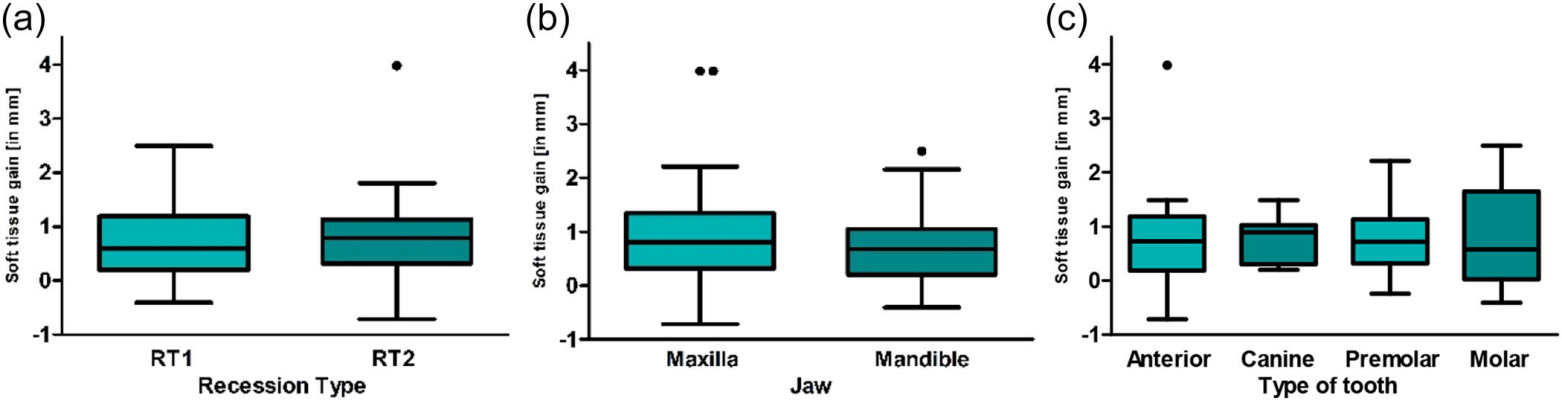
The mean absolute postoperative recession reduction is presented in millimeters (mm) based on (a) recession type, (b) jaw type, and (c) tooth type.

The mRR was 0.79 ± 0.73 mm. Regarding jaw types, a reduction of 0.73 ± 0.66 mm was observed in the maxilla, and a reduction of 0.84 ± 0.78 mm in the mandible (Figure 5). Concerning tooth types, absolute root coverage of 0.80 ± 0.95 mm was achieved for anterior teeth, 0.78 ± 0.43 mm for canines, 0.74 ± 0.56 mm for premolars, and 0.83 ± 0.88 mm for molars (Figure 5). For both recession types, a reduction of 0.76 ± 0.74 mm was observed for RT 1, and a reduction of 0.81 ± 0.72 mm for RT 2, 12 months postoperation. No statistical significance was found between recession type (*p* = 0.798), jaw type (*p* = 0.485), or tooth type (*p* = 0.976).

### 3.7 Gingival Thickness

**Figure 6:**
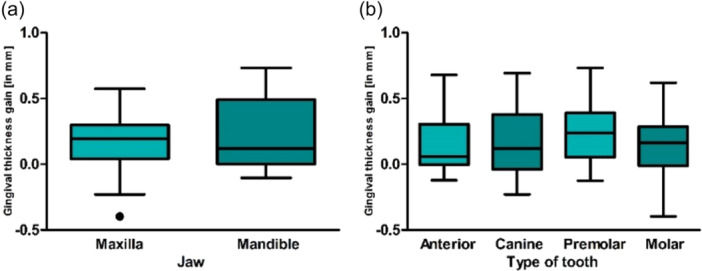
The mean postoperative gingival volume increase is presented as in millimeters (mm) based on (a) jaw type and (b) tooth type.

The gingival thickness of all recession coverages in the study increased by an average of 0.23 ± 0.24 mm over the course of 12 months postoperation. When analyzed by jaw location, a thickness gain of 0.26 ± 0.26 mm was observed in the mandible, compared to 0.18 ± 0.20 mm in the maxilla (Figure 6). Recession coverages classified as RT 1 showed a thickness gain of 0.17 ± 0.19 mm. For RT 2, a thickness gain of 0.28 ± 0.26 mm was recorded. The thickness gain at anterior teeth was 0.19 ± 0.26 mm, for canines it was 0.23 ± 0.18 mm, for premolars it was 0.24 ± 0.21 mm, and for molars, a gain of 0.22 ± 0.23 mm was achieved. Regarding volume increase, there was no significant difference observed among the results for RT classes (*p* = 0.297). However, a significant difference was observed between the jaws. With a significant volume increase in the mandible compared to the maxilla (*p* = 0.042).

### 3.8 Intrarater and Interrater Reproducibility

The intrarater reproducibility (rater 1, ICC of 0.997, rater 2, ICC of 0.995), demonstrated a high level of consistency. The overall interrater reproducibility of 0.999 highlights the strong agreement between raters (Hanney et al. 2011; Conejo Bayón et al. 2014).

## 4 Discussion

In the current study, a follow‐up period of 1 year revealed a mean root coverage (mRC) of 69.47% and a complete root coverage (cRC) of 29.87%, with complete root coverage achieved in 33.34% of the cases. These results are in line with previous randomized controlled trials (RCTs) that have reported 12‐month follow‐up mRC rates ranging from 53.2% to 73.2% and cRC rates from 20% to 40.74% in cases of Miller Class I and II recessions treated with various porcine dermal matrices in conjunction with the MCAT (Aroca et al. 2013; Vincent‐Bugnas et al. 2021; Cosgarea et al. 2016; Pietruska et al. 2019). Notably, an RCT focused on the treatment of Miller Class III recessions using a connective tissue graft (CTG) reported an mRC of 82% and a cRC of 40% over a 12‐month healing period (Aroca et al. 2010). In contrast, RCTs utilizing matrices such as allogenic dermal matrices (mRC: 95.5%, cRC: 72.7%; Ahmedbeyli et al. 2019) or porcine collagen matrices (mRC: 71%–85%, cRC: 42–46, 8%; Aroca et al. 2013; Rakasevic et al. 2020) have generally shown higher success rates, although in these studies, a periodontal Probe was used. Measurements are typically rounded to the nearest 0.5 mm, leading to deviations of up to 2.7 mm due to factors like angulation, pressure, and tissue inflammation (Kuralt et al. 2021). These inaccuracies reduce reliability and comparability across studies. In contrast, digital measurement methods offer higher precision, achieving accuracy levels of 0.01 mm with improved inter‐ and intra‐rater reliability (Fageeh et al. 2019; Dritsas et al. 2023). Digital techniques minimize errors associated with manual probing, providing more consistent and reliable results. However, they may have practical limitations such as cost and accessibility.

Despite these variations, autologous CTGs remain the gold standard for recession coverage in dental practice due to their high predictability and long‐term stability (Terzievska et al. 2023; Harris 2003) with enhanced esthetic outcomes and stable results over time (Tavelli et al. 2019). Interestingly, the present study did not reveal significant differences in coverage rates between the maxilla and mandible. This contrasts with findings from Aroca et al and Chambrone et al who observed higher coverage rates in the maxilla and noted a 0.94 mm lesser recession reduction in the mandible (Cosgarea et al. 2016; Chambrone et al. 2008; Aroca et al. 2018). In this study, there was no correlation between tooth type and recession coverage, even though the review of (Gil et al. 2018) revealed that molars, along with initial root prominence, interdental tissue loss, initial recession depth, and width were negatively correlated with the achievement of periodontal root coverage. The lack of jaw type influence in this study could be attributed to factors such as the surgical technique employed, the use of MCAT, and extraoral taping, which may have minimized differences between the two anatomical regions. Other studies have identified factors such as thicker papillary ridges, superior vascularization, facial muscle tension, and a shallow vestibule in the maxilla as contributing to more successful outcomes, while thinner gingival phenotypes, flap thickness, and mandibular tension can limit success in the mandible (Aroca et al. 2018; Chambrone and Chambrone 2006). In case of RT2 recessions, we routinely used a greater flap release including more adjacent teeth, which explains the contradiction of better results for RT2 recessions compared to existing literature.

Regarding gingival thickness, the present study observed a modest gain of 0.23 mm after 1 year, which is consistent with other recent reports showing gains ranging from 0.27 to 0.40 mm following treatment with porcine dermal matrices, measured transgingivally. In contrast, CTG procedures, using the same surgical approach, have been associated with more substantial gains in gingival thickness, ranging from 1 to 1.1 mm (Vincent‐Bugnas et al. 2021; Pietruska et al. 2019). The systematic review of (Cairo 2017) emphasized the critical role of gingival thickness in the success of root coverage procedures, highlighting its positive correlation with clinical outcomes. As such, transgingival measurement techniques, such as those using an endodontic file (Pietruska et al. 2019; Kaya et al. 2018) are vital for accurately gauging soft tissue gains. When comparing the 6‐month and 12‐month data from this study, it is evident that the clinical outcomes remained stable, with minimal changes in mRC, cRC, and horizontal gingival volume. Specifically, the mean root recession (mRR) for all teeth showed a slight decrease of only −0.03 mm (from 0.82 ± 0.78 mm to 0.79 ± 0.73 mm). The mRC values increased by +5% (from 63.4% ± 46.7% to 69.4% ± 61%) and the cRC by 1.3% (from 28.6% to 29.9%). Gingival volume also remained stable, with a minor decrease of 0.07 ± 0.24 mm. The precision of our measurements was further validated, with a low average deviation of 0.039 ± 0.007 mm when comparing preoperative and postoperative models. This deviation is smaller than the expected measurement inaccuracies associated with transgingival techniques, reinforcing the reliability of our data. The intrarater reproducibility (ICC of 0.99 vs. ICC of 0.995) demonstrated a high degree of consistency, which is essential for maintaining data integrity in studies with subjective assessments. Additionally, the interrater reproducibility (0.999) further attests to the robustness of the data, minimizing variability and enhancing the credibility of our findings (Koo and Li 2016).

Several limitations of the study should be noted. First, the prospective, single‐arm design of the study lacked a control group, which makes it challenging to draw definitive conclusions about the effects of the surgical intervention alone. Furthermore, the absence of randomization introduced a potential selection bias in the patient group. In an effort to match the 6‐month results from Iglhaut et al we used a plaster cast to generate impressions; however, this led to artefacts and the exclusion of some patient data. Future studies would benefit from fully digital workflows using intra‐oral scanners, which would mitigate such issues. The increasing adoption of intra‐oral scanner integrated workflows necessitates the development of standardized protocols, including consistent landmarks, uniform scanning systems, software, and CEJ‐independent measurements (Kuralt et al. 2021). The study's lack of keratinized tissue width (KTW) assessment limits its validity, as KTW is a critical parameter for long‐term stability of gingival margins around teeth and peri‐implant soft tissue health (Carbone et al. 2024). While tunneling procedures typically do not increase KTW, surgical interventions such as flap release or compromised vascularization may reduce it, potentially affecting long‐term outcomes (Nickles et al. 2010). It is important to distinguish that, around natural teeth, the clinical relevance of KTW remains a topic of debate; however, the 2015 consensus report of the American Academy of Periodontology suggested that ≥ 2 mm of keratinized tissue may be beneficial, particularly when plaque control is suboptimal (Scheyer et al. 2015). In contrast, a growing body of evidence indicates that an adequate width of keratinized mucosa (KMW) around dental implants plays a more decisive role in preventing plaque accumulation, mucosal inflammation, bleeding on probing, and buccal soft tissue recession (Ramanauskaite et al. 2022). Although the direct impact of KMW on peri‐implantitis in terms of probing depth or bone loss may be limited, a width of ≥ 2 mm is widely regarded as beneficial for ensuring soft tissue stability, reducing inflammation, and improving patient comfort during oral hygiene (Lin et al. 2013). Therefore, measurement of KTW and KMW should be considered essential in both periodontal and peri‐implant surgical planning. Unfortunately, this assessment is not yet feasible within fully digital workflows, representing a current limitation of digital measurement methodologies.

In conclusion, the clinical results of this study, following 12 months of treatment with a porcine dermal matrix for recession coverage, demonstrate stable mRC, cRC, and gingival volume outcomes. These findings are comparable to the 6‐month results, and the study provides valuable insights into the effectiveness of this treatment approach, irrespective of recession type, tooth type, or jaw type.

## 5 Conclusions

The present findings of this study indicate that the PDM in combination with the MCAT yields stable root coverage results for treating gingival recessions at 12 months postoperative.

## Author Contributions

*Supervision*: Tobias Fretwurst, Gerhard Iglhaut, and Katja Nelson. *Conceptualization*: Victoria Constanze Landwehr, Tobias Fretwurst, Gerhard Iglhaut, Katja Nelson, and Anton Sculean. *Methodology*: Victoria Constanze Landwehr, Tobias Fretwurst, Gerhard Iglhaut, Katja Nelson, and Anton Sculean. *Data collection*: Gerhard Iglhaut, Victoria Constanze Landwehr, Tobias Fretwurst, Sebastian Ollinge, and Erik Würflein. *Statistical analysis*: Kirstin Vach. *Interpretation of the data*: Erik Würflein, Victoria Constanze Landwehr, Tobias Fretwurst, Gerhard Iglhaut, Katja Nelson, Sebastian Ollinge, and Anton Sculean. *Drafting of the manuscript:* Erik Würflein, Victoria Constanze Landwehr, Tobias Fretwurst, and Gerhard Iglhaut. *Critical revision of the manuscript, review, and editing:* Erik Würflein, Victoria Constanze Landwehr, Tobias Fretwurst, Gerhard Iglhaut, Katja Nelson, Sebastian Ollinge, and Anton Sculean. *Final approval:* Erik Würflein, Victoria Constanze Landwehr, Kirstin Vach, Katja Nelson, Sebastian Ollinge, Anton Sculean, Gerhard Iglhaut, and Tobias Fretwurst.

## Ethics Statement

This study was approved by the ethics committee of the Faculty of Medicine at the University of Freiburg, Germany (No 352/19), and all participants provided written informed consent before their inclusion in the study.

## Consent

All authors have reviewed and approved the final version of the manuscript and consent to its publication.

## Conflicts of Interest

The authors declare no conflicts of interest.

## Supporting information


**Supporting Information:** Baseline Characteristics of Patients and Sites.

## Data Availability

The data that support the findings of this study are available from the corresponding author upon reasonable request.

## References

[cre270199-bib-0001] Ahmedbeyli, C. , S. D. Ipci , G. Cakar , and S. Yilmaz . 2019. “Laterally Positioned Flap Along With Acellular Dermal Matrix Graft in the Management of Maxillary Localized Recessions.” Clinical Oral Investigations 23, no. 2: 595–601. 10.1007/s00784-018-2475-1. 29725851

[cre270199-bib-0002] Aichelmann‐Reidy, M. E. , R. A. Yukna , G. H. Evans , H. F. Nasr , and E. T. Mayer . 2001. “Clinical Evaluation of Acellular Allograft Dermis for the Treatment of Human Gingival Recession.” Journal of Periodontology 72, no. 8: 998–1005. 10.1902/jop.2001.72.8.998. 11525450

[cre270199-bib-0003] Allen, E. P. 2010. “Subpapillary Continuous Sling Suturing Method for Soft Tissue Grafting With the Tunneling Technique.” International Journal of Periodontics and Restorative Dentistry 30, no. 5: 479–485. 20814601

[cre270199-bib-0004] De Annuntiis, C. , L. Testarelli , and R. Guarnieri . 2022. “Use of Xenogenic Collagen Matrices in Peri‐Implant Soft Tissue Volume Augmentation: A Critical Review on the Current Evidence and New Technique Presentation.” Materials 15, no. 11: 3937. 10.3390/ma15113937. https://mdpi-res.com/d_attachment/materials/materials-15-03937/article_deploy/materials-15-03937-v2.pdf?version=1654072527. 35683237 PMC9182004

[cre270199-bib-0005] Aroca, S. , A. Barbieri , M. Clementini , F. Renouard , and M. de Sanctis . 2018. “Treatment of Class III Multiple Gingival Recessions: Prognostic Factors for Achieving a Complete Root Coverage.” Journal of Clinical Periodontology 45, no. 7: 861–868. 10.1111/jcpe.12923. 29757468

[cre270199-bib-0006] Aroca, S. , T. Keglevich , D. Nikolidakis , et al. 2010. “Treatment of Class III Multiple Gingival Recessions: A Randomized‐Clinical Trial.” Journal of Clinical Periodontology 37, no. 1: 88–97. 10.1111/j.1600-051X.2009.01492.x. 19968743

[cre270199-bib-0007] Aroca, S. , B. Molnár , P. Windisch , et al. 2013. “Treatment of Multiple Adjacent Miller Class I and II Gingival Recessions With a Modified Coronally Advanced Tunnel (MCAT) Technique and a Collagen Matrix or Palatal Connective Tissue Graft: A Randomized, Controlled Clinical Trial.” Journal of Clinical Periodontology 40, no. 7: 713–720. 10.1111/jcpe.12112. 23627374

[cre270199-bib-0008] Cairo, F. 2017. “Periodontal Plastic Surgery of Gingival Recessions at Single and Multiple Teeth.” Periodontology 2000 75, no. 1: 296–316. 10.1111/prd.12186. 28758301

[cre270199-bib-0009] Cairo, F. , M. Nieri , S. Cincinelli , J. Mervelt , and U. Pagliaro . 2011. “The Interproximal Clinical Attachment Level to Classify Gingival Recessions and Predict Root Coverage Outcomes: An Explorative and Reliability Study.” Journal of Clinical Periodontology 38, no. 7: 661–666. 10.1111/j.1600-051X.2011.01732.x. 21507033

[cre270199-bib-0010] Cairo, F. , M. Nieri , and U. Pagliaro . 2014. “Efficacy of Periodontal Plastic Surgery Procedures in the Treatment of Localized Facial Gingival Recessions. A Systematic Review.” Journal of Clinical Periodontology 41, no. s15: S44–S62. 10.1111/jcpe.12182. 24641000

[cre270199-bib-0011] Cairo, F. , U. Pagliaro , and M. Nieri . 2008. “Treatment of Gingival Recession With Coronally Advanced Flap Procedures: A Systematic Review.” Journal of Clinical Periodontology 35, no. s8: 136–162. 10.1111/j.1600-051X.2008.01267.x. 18724847

[cre270199-bib-1001] Cairo, F. , R. Rotundo , P. D. Miller , and G. P. Pini Prato . 2009. “Root Coverage Esthetic Score: A System to Evaluate the Esthetic Outcome of the Treatment of Gingival Recession Through Evaluation of Clinical Cases.” Journal of Periodontology 80, no. 4: 705–710. 10.1902/jop.2009.080565. 19335093

[cre270199-bib-0012] Carbone, A. C. , J. C. Joly , J. Botelho , et al. 2024. “Long‐Term Stability of Gingival Margin and Periodontal Soft‐Tissue Phenotype Achieved After Mucogingival Therapy: A Systematic Review.” Journal of Clinical Periodontology 51, no. 2: 177–195. 10.1111/jcpe.13900. 37963451

[cre270199-bib-0013] Carvalho, P. F. M. , R. C. da Silva , P. R. Cury , and J. C. Joly . 2006. “Modified Coronally Advanced Flap Associated With a Subepithelial Connective Tissue Graft for the Treatment of Adjacent Multiple Gingival Recessions.” Journal of Periodontology 77, no. 11: 1901–1906. 10.1902/jop.2006.050450. 17076617

[cre270199-bib-0014] Chambrone, L. , R. C. N. de Castro Pinto , and L. A. Chambrone . 2019. “The Concepts of Evidence‐Based Periodontal Plastic Surgery: Application of the Principles of Evidence‐Based Dentistry for the Treatment of Recession‐Type Defects.” Periodontology 2000 79, no. 1: 81–106. 10.1111/prd.12248. 30892767

[cre270199-bib-0015] Chambrone, L. , D. Chambrone , F. E. Pustiglioni , L. A. Chambrone , and L. A. Lima . 2008. “Can Subepithelial Connective Tissue Grafts Be Considered the Gold Standard Procedure in the Treatment of Miller Class I and II Recession‐Type Defects?” Journal of Dentistry 36, no. 9: 659–671. 10.1016/j.jdent.2008.05.007. 18584934

[cre270199-bib-0016] Chambrone, L. A. , and L. Chambrone . 2006. “Subepithelial Connective Tissue Grafts in the Treatment of Multiple Recession‐Type Defects.” Journal of Periodontology 77, no. 5: 909–916. 10.1902/jop.2006.050249. 16671886

[cre270199-bib-0017] Conejo Bayón, F. , J. Maese , A. Fernandez Oliveira , et al. 2014. “Feasibility of the Medial Temporal Lobe Atrophy Index (MTAi) and Derived Methods for Measuring Atrophy of the Medial Temporal Lobe.” Frontiers in Aging Neuroscience 6: 305. 25414666 10.3389/fnagi.2014.00305 PMC4220710

[cre270199-bib-0018] Cosgarea, R. , R. Juncar , C. Heumann , et al. 2016. “Non‐surgical Periodontal Treatment in Conjunction With 3 or 7 Days Systemic Administration of Amoxicillin and Metronidazole in Severe Chronic Periodontitis Patients. A Placebo‐Controlled Randomized Clinical Study.” Journal of Clinical Periodontology 43, no. 9: 767–777. 10.1111/jcpe.12559. 27027501

[cre270199-bib-1002] Di Gianfilippo, R. , I. C. Wang , L. Steigmann , D. Velasquez , H.‐L. Wang , and H.‐L Chan . 2021. “Efficacy of Microsurgery and Comparison to Macrosurgery for Gingival Recession Treatment: A Systematic Review With Meta‐Analysis.” Clinical Oral Investigations 25, no. 7: 4269–4280. 10.1007/s00784-021-03954-0. 33928441

[cre270199-bib-0019] Dritsas, K. , D. Halazonetis , M. Ghamri , A. Sculean , C. Katsaros , and N. Gkantidis . 2023. “Accurate Gingival Recession Quantification Using 3D Digital Dental Models.” Clinical Oral Investigations 27, no. 4: 1697–1705. 10.1007/s00784-022-04795-1. 36424472 PMC10102060

[cre270199-bib-0020] Fageeh, H. N. , A. A. Meshni , H. A. Jamal , R. S. Preethanath , and E. Halboub . 2019. “Correction To: The Accuracy and Reliability of Digital Measurements of Gingival Recession Versus Conventional Methods.” BMC Oral Health 19, no. 1: 199. 10.1186/s12903-019-0876-4. 31470838 PMC6716887

[cre270199-bib-0021] Gil, A. , N. Bakhshalian , S. Min , and H. H. Zadeh . 2018. “Treatment of Multiple Recession Defects With Vestibular Incision Subperiosteal Tunnel Access (VISTA): A Retrospective Pilot Study Utilizing Digital Analysis.” Journal of Esthetic and Restorative Dentistry 30, no. 6: 572–579. 10.1111/jerd.12434. 30367715

[cre270199-bib-0022] Hanney, W. J. , M. J. Kolber , and J. S. Marshall . 2011. “The Reliability of Clinical Measurements Designed to Quantify Shoulder Mobility.” Physical Therapy Reviews 16, no. 6: 413–422. 10.1179/1743288X11Y.0000000023.

[cre270199-bib-0023] Harris, R. J. 2003. “Root Coverage in Molar Recession: Report of 50 Consecutive Cases Treated With Subepithelial Connective Tissue Grafts.” Journal of Periodontology 74, no. 5: 703–708. 10.1902/jop.2003.74.5.703. 12816304

[cre270199-bib-0024] Iglhaut, G. , T. Fretwurst , L. Schulte , et al. 2024. “Digital Workflow to Assess Gingival Recession Coverage Independently of the Cemento–Enamel Junction: A Prospective Clinical Study Using the Modified Coronally Advanced Tunnel Technique With Porcine Dermal Matrix.” Clinical Oral Investigations 28, no. 11: 613. 10.1007/s00784-024-05936-4. 39463191 PMC11513702

[cre270199-bib-0025] Joss‐Vassalli, I. , C. Grebenstein , N. Topouzelis , A. Sculean , and C. Katsaros . 2010. “Orthodontic Therapy and Gingival Recession: A Systematic Review.” Orthodontics & Craniofacial Research 13, no. 3: 127–141. 10.1111/j.1601-6343.2010.01491.x. 20618715

[cre270199-bib-0026] Kassab, M. M. , H. Badawi , and A. R. Dentino . 2010. “Treatment of Gingival Recession.” Dental Clinics of North America 54, no. 1: 129–140. 10.1016/j.cden.2009.08.009. 20103476

[cre270199-bib-0027] Kassab, M. M. , and R. E. Cohen . 2003. “The Etiology and Prevalence of Gingival Recession.” Journal of the American Dental Association 134, no. 2: 220–225. 10.14219/jada.archive.2003.0137. 12636127

[cre270199-bib-0028] Kaya, Y. , Ö. Alkan , E. A. Alkan , and S. Keskin . 2018. “Gingival Thicknesses of Maxillary and Mandibular Anterior Regions in Subjects With Different Craniofacial Morphologies.” American Journal of Orthodontics and Dentofacial Orthopedics 154, no. 3: 356–364. 30173838 10.1016/j.ajodo.2017.11.039

[cre270199-bib-0029] Koo, T. K. , and M. Y. Li . 2016. “A Guideline of Selecting and Reporting Intraclass Correlation Coefficients for Reliability Research.” Journal of Chiropractic Medicine 15, no. 2: 155–163. 10.1016/j.jcm.2016.02.012. 27330520 PMC4913118

[cre270199-bib-0030] Kuralt, M. , R. Gašperšič , and A. Fidler . 2021. “The Precision of Gingival Recession Measurements Is Increased by an Automated Curvature Analysis Method.” BMC Oral Health 21: 505. 10.1186/s12903-021-01858-9. 34620155 PMC8499415

[cre270199-bib-0031] Lin, G.‐H. , H.‐L. Chan , and H.‐L. Wang . 2013. “The Significance of Keratinized Mucosa on Implant Health: A Systematic Review.” Journal of Periodontology 84, no. 12: 1755–1767. 10.1902/jop.2013.120688. 23451989

[cre270199-bib-0032] Lin, Z. , C. Nica , A. Sculean , and M. B. Asparuhova . 2020. “Enhanced Wound Healing Potential of Primary Human Oral Fibroblasts and Periodontal Ligament Cells Cultured on Four Different Porcine‐Derived Collagen Matrices.” Materials 13, no. 17: 3819. 10.3390/ma13173819. 32872458 PMC7504420

[cre270199-bib-0033] Merijohn, G. K. 2016. “Management and Prevention of Gingival Recession.” Periodontology 2000 71, no. 1: 228–242. 10.1111/prd.12115. 27045439

[cre270199-bib-0034] Miranda‐Rius, J. , L. Brunet‐Llobet , and E. Lahor‐Soler . 2018. “The Periodontium as a Potential Cause of Orofacial Pain: A Comprehensive Review.” Open Dentistry Journal 12: 520–528. 10.2174/1874210601812010520. 30197691 PMC6110068

[cre270199-bib-0035] Moustafa, M. , H. Abdel Gaber , and R. Hussein . 2022. “Xenogeneic Acellular Dermal Matrix Versus Connective Tissue Graft in Conjunction With Tunneling Technique in Treatment of Gingival Recession (Randomized Controlled Clinical Trial).” Egyptian Dental Journal 68, no. 4: 3391–3400. 10.21608/edj.2022.160170.2240.

[cre270199-bib-0036] Nickles, K. , P. Ratka‐Krüger , E. Neukranz , P. Raetzke , and P. Eickholz . 2010. “Ten‐Year Results After Connective Tissue Grafts and Guided Tissue Regeneration for Root Coverage.” Journal of Periodontology 81, no. 6: 827–836. 10.1902/jop.2010.090632. 20450359

[cre270199-bib-0037] Pietruska, M. , A. Skurska , Ł. Podlewski , R. Milewski , and J. Pietruski . 2019. “Clinical Evaluation of Miller Class I and II Recessions Treatment With the Use of Modified Coronally Advanced Tunnel Technique With Either Collagen Matrix or Subepithelial Connective Tissue Graft: A Randomized Clinical Study.” Journal of Clinical Periodontology 46, no. 1: 86–95. 10.1111/jcpe.13031. 30362599

[cre270199-bib-0038] Rakasevic, D. L. , I. Z. Milinkovic , S. M. Jankovic , I. A. Soldatovic , Z. M. Aleksic , and N. S. Nikolic‐Jakoba . 2020. “The Use of Collagen Porcine Dermal Matrix and Connective Tissue Graft With Modified Coronally Advanced Tunnel Technique in the Treatment of Multiple Adjacent Type I Gingival Recessions: A Randomized, Controlled Clinical Trial.” Journal of Esthetic and Restorative Dentistry 32, no. 7: 681–690. 10.1111/jerd.12624. 32706184

[cre270199-bib-0039] Ramanauskaite, A. , F. Schwarz , and R. Sader . 2022. “Influence of Width of Keratinized Tissue on the Prevalence of Peri‐Implant Diseases: A Systematic Review and Meta‐Analysis.” Clinical Oral Implants Research 33, no. S23: 8–31. 10.1111/clr.13766. 35763022

[cre270199-bib-0040] Rebele, S. F. , O. Zuhr , D. Schneider , R. E. Jung , and M. B. Hürzeler . 2014. “Tunnel Technique With Connective Tissue Graft Versus Coronally Advanced Flap With Enamel Matrix Derivative for Root Coverage: A RCT Using 3D Digital Measuring Methods. Part II. Volumetric Studies on Healing Dynamics and Gingival Dimensions.” Journal of Clinical Periodontology 41, no. 6: 593–603. 10.1111/jcpe.12254. 24708338

[cre270199-bib-0041] Scheyer, E. T. , M. Sanz , S. Dibart , et al. 2015. “Periodontal Soft Tissue Non‐root Coverage Procedures: A Consensus Report From the AAP Regeneration Workshop.” Journal of Periodontology 86, no. 2S: 73–76. 10.1902/jop.2015.140377. 25644301

[cre270199-bib-0042] Sculean, A. , R. Gruber , and D. D. Bosshardt . 2014. “Soft Tissue Wound Healing Around Teeth and Dental Implants.” Journal of Clinical Periodontology 41, no. s15: S6–S22. 10.1111/jcpe.12206. 24641001

[cre270199-bib-0043] Sculean, A. , R. Cosgarea , A. Stähli , et al. 2016. “Treatment of Multiple Adjacent Maxillary Miller Class I, II, and III Gingival Recessions With the Modified Coronally Advanced Tunnel, Enamel Matrix Derivative, and Subepithelial Connective Tissue Graft: A Report of 12 Cases.” Quintessence International: 653–659. 10.3290/j.qi.a36562. 27446995

[cre270199-bib-0044] Sculean, A. , C. Raluca , C. Katsaros , et al. 2017. “Treatment of Single and Multiple Miller Class I and III Gingival Recessions at Crown‐Restored Teeth in Maxillary Esthetic Areas.” Quintessence International: 772–782. 10.3290/j.qi.a39031. 28944378

[cre270199-bib-0045] Tavelli, L. , S. Barootchi , H. Greenwell , and H.‐L. Wang . 2019. “Is a Soft Tissue Graft Harvested From the Maxillary Tuberosity the Approach of Choice in An Isolated Site?” Journal of Periodontology 90, no. 8: 821–825. 10.1002/JPER.18-0615. 30690733

[cre270199-bib-0046] Terzievska, A. , D. Veleska‐Stevkovska , G. Apostolova , Z. Mencheva , and S. Trajculeski . 2023. “The Effectiveness of the Influence of the Second Generation Platelet‐Rich Fibrin in the Treatment of Localized Individual Miller I and II Gingival Recessions (Case Report).” South East European Journal of Immunology 6, no. 1: 62–69. 10.3889/seejim.2023.6038.

[cre270199-bib-0047] Vincent‐Bugnas, S. , J. Laurent , E. Naman , M. Charbit , and G. Borie . 2021. “Treatment of Multiple Gingival Recessions With Xenogeneic Acellular Dermal Matrix Compared to Connective Tissue Graft: A Randomized Split‐Mouth Clinical Trial.” Journal of Periodontal & Implant Science 51, no. 2: 77–87. 10.5051/jpis.2002400120. 33913631 PMC8090794

[cre270199-bib-0048] Xue, F. , R. Zhang , Y. Zhang , et al. 2022. “Treatment of Multiple Gingival Recessions With Concentrated Growth Factor Membrane and Coronally Advanced Tunnel Technique via Digital Measurements: A Randomized Controlled Clinical Trial.” Journal of Dental Sciences 17, no. 2: 725–732. 10.1016/j.jds.2021.10.012. 35756792 PMC9201548

[cre270199-bib-0049] Zhan, X. , W. Yan , J. Yan , W. Tong , W. Chen , and Y. Lin . 2021. “LPCGF and EDTA Conditioning of the Root Surface Promotes the Adhesion, Growth, Migration and Differentiation of Periodontal Ligament Cells.” Journal of Periodontology 92, no. 5: 738–747. 10.1002/JPER.20-0103. 32835432

[cre270199-bib-0050] Zucchelli, G. , and I. Mounssif . 2015. “Periodontal Plastic Surgery.” Periodontology 2000 68, no. 1: 333–368. 10.1111/prd.12059. 25867992

[cre270199-bib-0051] Zuhr, O. , D. Akakpo , P. Eickholz , K. Vach , M. B. Hürzeler , and H. Petsos . 2021. “Tunnel Technique With Connective Tissue Graft Versus Coronally Advanced Flap With Enamel Matrix Derivate for Root Coverage: 5‐Year Results of an RCT Using 3D Digital Measurement Technology for Volumetric Comparison of Soft Tissue Changes.” Journal of Clinical Periodontology 48, no. 7: 949–961. 10.1111/jcpe.13470. 33847022

