# Supplementary material for: Modified Coronally Advanced Tunnel Technique With Porcine Dermal Matrix for Recession Treatment: 12‐Month Follow‐Up
Source: Clin Exp Dent Res. 2025 Aug 22;11(5):e70199. doi: 10.1002/cre2.70199 (PMC12371723; doi:10.1002/cre2.70199)
Supplement: Supplementary file 1 — Supporting Information: Baseline Characteristics of Patients and Sites. [file CRE2-11-e70199-s001.docx]

| **Parameter** |  |
| --- | --- |
| Patient number (n) | 19 |
| Teeth (n) | 77 |
| Age (mean ± SD) (years) | 46.09 ± 14.94 |
| Gender (Female/Male) (n) | 15/4 |
| Gingival recession Type (n) | RT 1: 34 RT 2: 43 |
| Maxillary/mandibular sites (n) | 35/42 |
| Maxillary sites (n)  Central/lateral incisor sites (n)  Canine sites (n)  Premolar sites (n)  Molar sites (n)  Mandibular sites (n)  Central/lateral incisor sites (n)  Canine sites (n)  Premolar sites (n)  Molar sites (n) | 35  6  7  11  11  42  13  6  16  7 |
| Recession depth (mean ± SD) (mm) | 1.26 ± 0.86 |

**Supplementary material:** Baseline Characteristics of Patients and Sites
